# Multi-center study of inter-rater reproducibility, image quality, and diagnostic accuracy of CZT versus conventional SPECT myocardial perfusion imaging

**DOI:** 10.1007/s12350-022-03054-w

**Published:** 2022-07-07

**Authors:** Nabeel Mansour, Stephan G. Nekolla, Eliana Reyes, George Angelidis, Panagiotis Georgoulias, Constantinos Anagnostopoulos, Paco Bravo, Isabella Bruno, Albert Flotats, Francisco Fuentes-Ocampo, Roberto Sciagrà, Felix Keng, Lukas Kessler, Maria Papathanasiou, Prem Soman, Christoph Rischpler

**Affiliations:** 1 Department of Nuclear Medicine, Klinikum Rechts der Isar, Technical University Munich, Munich, Germany; 2 Department of Radiology, University Hospital, LMU Munich, Marchioninistr. 15, 81377 Munich, Germany; 3 DZHK (German Centre for Cardiovascular Research), Partner Site Munich Heart Alliance, Munich, Germany; 4 Royal Brompton and Harefield NHS Trust, London, UK; 5 Department of Nuclear Medicine, Faculty of Medicine, University of Thessaly, University Hospital of Larissa, Larissa, Greece; 6 BRFAA – Biomedical Research Foundation Academy of Athens, Athens, Greece; 7 Divisions of Nuclear Medicine and Cardiology, Departments of Radiology and Medicine, Hospital of the University of Pennsylvania, Philadelphia, PA USA; 8 Fondazione Policlinico Universitario Agostino Gemelli IRCCS, Roma, Italy; 9 Nuclear Medicine Department, Hospital de la Santa Creu i Sant Pau, Universitat Autònoma de Barcelona, Barcelona, Spain; 10 Department of Experimental and Clinical Biomedical Sciences ‘Mario Serio’, University of Florence, Florence, Italy; 11 National Heart Centre, Singapore, Singapore; 12 Department of Cardiology, West German Heart and Vascular Center, University Hospital, Essen, Germany; 13 Division of Cardiology, University of Pittsburgh Medical Center, Pittsburgh, PA USA; 14 Department of Nuclear Medicine, University Hospital Essen, University of Duisburg-Essen, Essen, Germany

**Keywords:** cadmium-zinc-telluride, myocardial perfusion imaging, coronary artery disease, single-photon emission tomography

## Abstract

**Background:**

Cadmium-zinc-telluride (CZT)-based detectors exhibit higher diagnostic sensitivity in myocardial perfusion imaging (MPI) than conventional Anger-MPI for detection of coronary artery disease (CAD); however, reduced specificity and diagnostic accuracy of CZT-MPI were observed. This study aims to compare these different camera systems and to examine the degree of inter-rater reproducibility among readers with varying experience in MPI.

**Methods:**

83 patients who underwent double stress/rest examinations using both a CZT and conventional SPECT cameras within one visit were included. Anonymized and randomized MPI-images were distributed to 15 international readers using a standardized questionnaire. Subsequent coronary angiography findings of ten patients served as a reference for analysis of sensitivity and specificity.

**Results:**

Image quality was significantly better in CZT-MPI with significantly lower breast attenuation (*P *< 0.05). CZT-MPI exhibited higher sensitivity than Anger-MPI (87.5% vs. 62.5%) and significantly reduced specificity (40% vs. 100%). Readers experienced with both camera systems had the highest inter-rater agreement indicating higher reproducibility (CZT 0.54 vs. conv. 0.49, *P* < 0.05).

**Conclusions:**

Higher diagnostic sensitivity of CZT-MPI offers advantages in detection of CAD yet potentially of at the cost of reduced specificity, therefore it requires special training and a differentiated evaluation approach, especially for non-experienced readers with such camera systems.

**Supplementary Information:**

The online version contains supplementary material available at 10.1007/s12350-022-03054-w.

## Introduction

Myocardial perfusion imaging (MPI) is one of the most often used techniques worldwide for the detection and assessment of coronary artery disease (CAD). The diagnostic performance of conventional single-photon emission computed tomography (Anger-SPECT) is well established, with current data yielding estimates for sensitivity ranging between 0.82 and 0.91, and for specificity between 0.70 to 0.90, depending on clinical history and the cutoff chosen for significant CAD.^1^ In the last decade, rapid-acquisition dedicated cardiac camera systems with cadmium-zinc-telluride (CZT)-based detectors have been introduced into clinical practice.^2–6^ CZT-detectors were selected because of their superior energy resolution compared to sodium iodide (NaI (Tl)) detectors used in conventional Anger cameras.^7^ The CZT-based camera used in this study (D-SPECT; Spectrum Dynamics, Israel) is equipped with tungsten parallel-hole collimators, which show a significant improvement in geometric efficiency compared to standard lead parallel-hole low-energy, high-resolution (LEHR) collimators used in Anger cameras.^5^ Additionally, the open design of the CZT camera and the flexibility of the L-shaped detector columns enable better accessibility and acquisition in a semi-supine position which serves as an advantage for large sized patients or patients with orthopnea. However, this comes potentially at the cost of reduced specificity in obese patients examined in this position, as argued in multiple studies.^8,9^

In contrary to Anger-MPI, the ability to image upright and therefore the tendency for imaging of a mainly obese patients may result in reduced diagnostic specificity with inferolateral segments still suffering marked soft tissue attenuation on CZT cameras.^9^ Furthermore, most dedicated CZT cameras for MPI are not equipped with an integrated computed tomography (CT) component with most studies performed without attenuation correction (AC), harboring the risk of attenuation artifacts being interpreted as perfusion abnormalities.^10^

With the introduction of high speed solid-state cardiac cameras first studies comparing these technologies suggested high correlation in image quality compared to conventional SPECT, with an equivalent level of diagnostic confidence.^11–13^ In a meta-analysis performed by Nudi et al.^8^, CZT-MPI presented a relatively high rate of false positive results and reduced specificity particularly in imaging of obese patients, respectively. In the majority of the studies included, patient cohorts were paired based on specific clinical characteristics.

The objective of this study was to examine inter-rater reproducibility, diagnostic certainty, reader experience effects between CZT-MPI and conventional Anger-MPI in the same patient cohort in a multi-center analysis with readers of varying experience in the assessment of SPECT-MPI. In addition, we delineated whether and to what extent the two camera systems, differ in terms of image quality and susceptibility to artifacts.

## Methods

### Study design

Retrospective multicentric reading study with 15 international readers from the field of nuclear cardiology with varying experience in MPI. The readers evaluated anonymized and randomized MPI findings of 83 patients acquired with a CZT-based as well as with one of two conventional Anger SPECT cameras (total of 166 cases). Informed consent was waived due to the retrospective character of the study.

### Patients

Of the 361 patients who underwent myocardial perfusion imaging (MPI) in the year 2013 as part of the clinical routine in the Klinikum rechts der Isar, Munich, Germany, we identified 83 patients who were sequentially examined with a CZT (D-SPECT, Spectrum Dynamics) and with one of the two available conventional SPECT cameras (Siemens E.CAM or Symbia T6, Siemens Healthineers) within one visit both after stress and at rest. Data were originally collected in routine clinical practice at our institute when the CZT camera was newly introduced and were used for comparison with the conventional SPECTs to familiarize the readers with this new technology. The pretest probability for the presence or absence of significant obstructive CAD, defined as a ≥ 70% stenosis of at least one coronary artery was calculated using a consortium score to validate the indication for non-invasive diagnostics.^14^ In a proportion of patients, prior coronary angiographic data indicated the extent of vessel involvement (N = 41). Other patients received a subsequent coronary angiography depending on symptoms and the results of the non-invasive diagnostic work-up. Relevant coronary angiography results, defined as results acquired no later than 90 days after MPI, were available in ten patients. As part of the clinical routine, cardiovascular risk factors (arterial hypertension, diabetes mellitus, dyslipidemia, nicotine abuse, and history of nicotine abuse) were present in 64 patients.

### Readers

15 international readers from the field of nuclear cardiology with varying experience in the interpretation of SPECT-MPI were recruited. They were divided based on underlying experience into three groups of five participants each: Five readers with experience with both conventional SPECT cameras and a CZT-SPECT camera (group 1); five readers who only have experience with conventional SPECT cameras (group 2) and five readers with little or no experience in MPI interpretation (group 3). The anonymized and randomized MPI images were distributed in the form of static images of the short axes and horizontal/vertical long axes as well as the polar maps by means of an online study. The online study was carried out using Evasys Survey Grid^15^ with an encrypted invitation letter containing a Uniform Resource Locator (URL) address with an individual transaction number (TAN) password for each reader. A standardized questionnaire broadly used in the interpretation of MPI with questions regarding image quality, artifacts, final diagnosis, diagnostic certainty and the 17-segment model of the American Heart Association (AHA)^16^ was used. This standardized questionnaire is used for purely image-based evaluation of data, which make up the main building blocks of MPI findings. No clinical information was provided, and all readers were blinded to the modality of examination (CZT- or conventional SPECT-MPI).

### SPECT cameras

The recorded light pulses are amplified with photoelectron multipliers (PMT) that deliver electrical pulses proportional to photon energy. In this way, the activity of the radiotracer in the examined organ is visualized as a projection onto a plane. The gamma camera, which rotates around the patient and depicts isotope activity in multiple angle projections, enables the three-dimensional reconstruction of isotope activity and distribution in the body. Two conventional cameras were used in this study (Siemens E.CAM and Symbia T, Siemens Medical Solutions). A D-SPECT system (Spectrum Dynamics, Israel) was used for CZT-MPI.^17^ This system uses pixilated CZT crystal detector columns mounted in nine vertical columns that are arranged in a 90 ° geometry. No attenuation correction (AC) was applied in this study, since the D-SPECT camera used at the time of the study did not support it.

### SPECT protocols

79 patients were examined via one-day protocol and four patients via two-day protocol. The patients with the one-day protocol were examined sequentially with both camera technologies, first in stress and then in rest (66 times CZT-stress-MPI first and 13 times conventional stress MPI first). Mean time interval between the stress examinations was approximately 33 (± 31) minutes when CZT-MPI was performed as first procedure vs. 58 (± 15) minutes when Anger-MPI was performed as first procedure.

Injected activities of the perfusion tracer Tc-99m sestamibi were body-weight adjusted according to current guidelines^18^ resulting in mean dose of 414 (± 221) MBq for the stress study and 838 (± 282) MBq for the rest study in the one-day protocol. Using the two-day-protocol injected activities were 518 (± 255) MBq (stress) and 548 (± 230) MBq (rest), respectively. On conventional SPECT cameras, all patients were imaged in a supine position and on the CZT-SPECT system all patients were imaged using the standard semi-supine position.

Scan durations were 20 min fixed per scan in the conventional cameras and on average 12 (stress) resp. 5 (rest) minutes the D-SPECT using count limits.

### Image reconstruction and interpretation

Analysis of the volumetric data on the regional tracer distribution resulting from MPI was performed with a dedicated software (MunichHeart).^19^ This quantitative analysis software enables the quantification and thus the objectification of tracer accumulation in the heart both at rest and under stress. It has been validated in multiple phantom studies and in several data sets of healthy patients and patients with CAD of various degrees of severity.^20^ After volumetric data analysis and image reconstruction, radiotracer activity is assessed in sections along the short and long axes of the left ventricle. Furthermore, a polar map of the radiotracer distribution throughout the left ventricle is generated.

**Figure 1:**
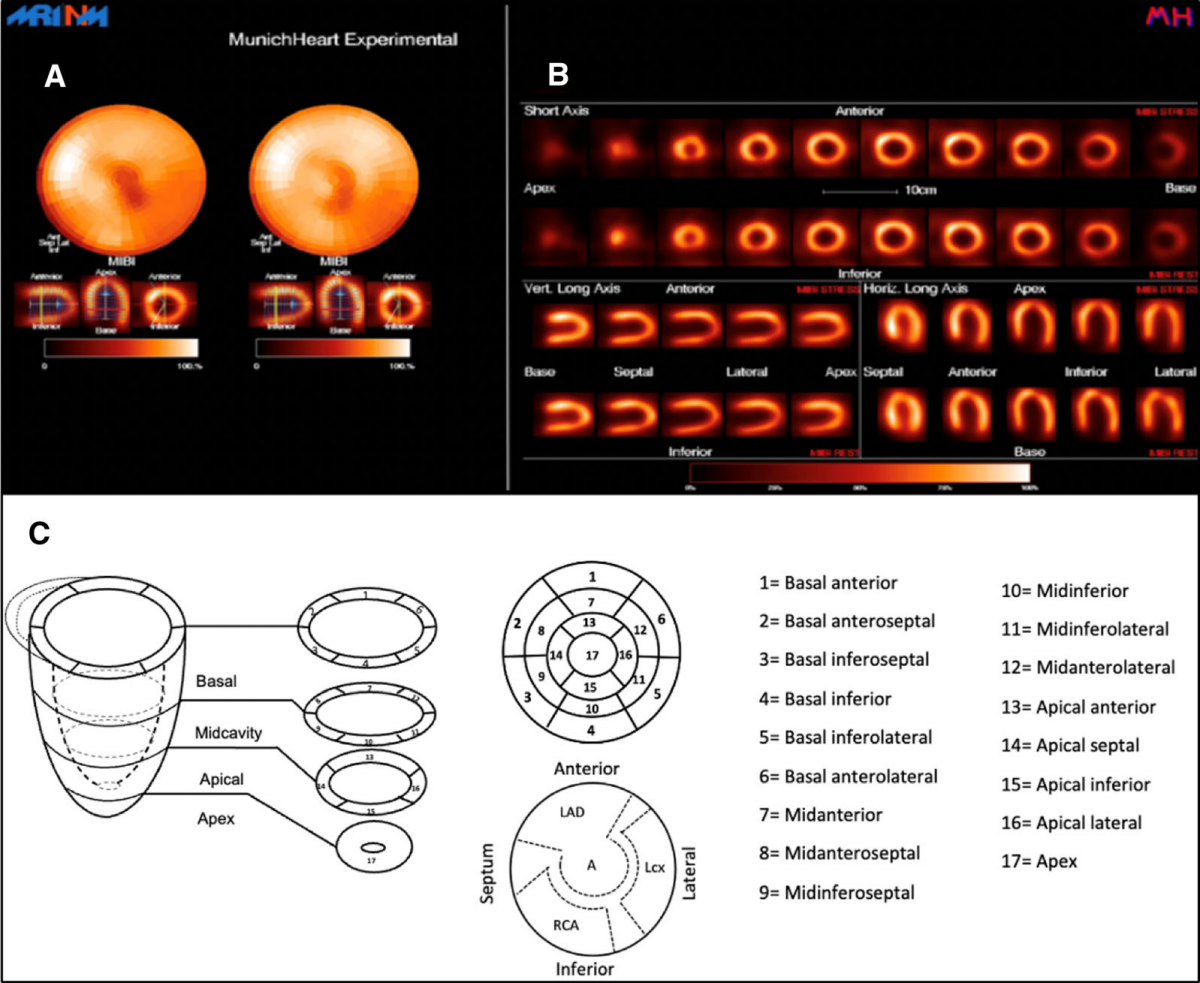
Myocardial perfusion imaging acquired with a D-SPECT camera. **A** Polarmaps of the stress- (left) and rest examination (right). **B** Static images of the short axes and horizontal/vertical long axes. C: Schematic illustration of the 17-segment model of the left ventricle with illustration of supply areas of the coronary arteries. *LAD* left anterior descending artery, *RCA* right coronary artery, *LCX* ramus circumflexus

A standardized myocardial scoring system for the resulting polar maps is available for quantification of results (summed stress score (SSS), summed rest score (SRS), and summed difference score (SDS) as described by Berman et al.^16^. As displayed in Figure 1, different segments represent different areas covered by each of the main coronary artery branches. Slight variations in allocation of the segments depending on coronary artery dominance can be observed.

### Statistical analysis

Statistical analysis was performed using R statistical software (R version 3.6.3). The Wilcoxon test was used to test differences between the two camera technologies or between two groups of readers.^21^ When individual values occurred very frequently, the sign test was calculated.^22^ Statistical significance was denoted by *P* values < .05. Inter-rater agreement was analyzed with the Fleiss' Kappa statistics.^23^ The analysis of the Inter-rater agreement was divided in two categories, the first regarding all answers for diagnosis (normal, ischemia, mixed ischemia/scar and scar), and the second regarding abnormality in comparison of normal versus abnormal findings (normal versus all other answers). Sensitivity, specificity, and accuracy were calculated using the angiography group (N = 10) and a negative control group which did not undergo angiography after non-invasive exclusion of CAD (N = 8). A positive finding was defined at a cutoff of ≥ 70% luminal diameter narrowing of at least one coronary vessel. Eight patients of the angiography group displayed coronary artery stenosis with the cutoff as defined above and two patients had normal angiograms. A total of ten patients were defined as a negative group consisting of patients with no relevant coronary artery stenosis in coronary angiography (N = 2) as well as patients who had intermediate pretest probability for CAD, yet no indication for a coronary angiography after revision of all non invasive examinations and available data and exclusion of CAD in interdisciplinary consensus (N = 8).

The individual coronary territories were compared in accordance with the 17-segment model of the AHA. A positive MPI finding was defined as an SDS of at least 2 and/or an SSS of at least 4. The SRS was assessed analogously to the SSS.

## Results

### Study population

83 patients aged between 38 and 91 years (mean, 67.7 ± 11.6) were enrolled. 49 patients (59%) were male. The body mass index (BMI) was between 16.5 and 55.4 kg/m^2^. Cardiovascular risk factors were documented only in 64 patients and 41 patients had pre-existing CAD as summarized in Table 1. Ten patients had coronary angiography results relevant to this study defined as results acquired within a maximum period of 90 days after MPI (median 14, range of 4 to 85 days). Coronary angiography revealed significant coronary artery stenosis, defined as ≥70% stenosis of vessel lumen in eight patients, of whom 7 received percutaneous transluminal coronary angioplasty (PTCA) and stenting. In one patient, a ≥ 70% stenosis was not revascularized due to an unsuccessful recanalization attempt of the occluded vessel. CAD was excluded in two patients with normal angiograms.

**Table 1:** Patient characteristics and cardiological history

Mean age (years)	67.7 ± 11.6
Male	49 (59%)
Female	34 (41%)
Cardiovascular risk factors (N=64)	
Hypertension	57 (89%)
Diabetes mellitus	18 (28%)
Dyslipidemia	43 (67%)
Smoking	12 (19%)
History of smoking	17 (27%)
Not documented	19 (23%)
Induced myocardial stress	
Exercise	20 (24%)
Adenosine	52 (63%)
Dobutamine	3 (4%)
Regadenoson	3 (4%)
Not documented	5 (6%)
Cardiological history (N=41)	
Number of diseased vessels	
0-vessel disease	20
1-vessel disease	4
2-vessel disease	5
3-vessel disease	12
Mean Agatston score	334.2
History of myocardial infarction	19 (46%)

		Mean	SD	Median	Min	Max
Tc-99m-MIBI Dose [MBq]	Rest	838.0	282.2	881.0	199	1404
	Stress	414.4	221.0	337.5	180	1108
EF [%]	Rest	60.0	11.6	60.0	23	80
	Stress	59.2	12.1	59.0	24	87

Cardiovascular risk factors were documented in 64 patients. 41 patients had a positive cardiological history regarding CAD

*Tc-99m* technetium-99m, *MIBI* sestamibi, *MBq* megabecquerel, *EF* ejection fraction, *SD* standard deviation

### Image quality and artifacts

**Figure 2:**
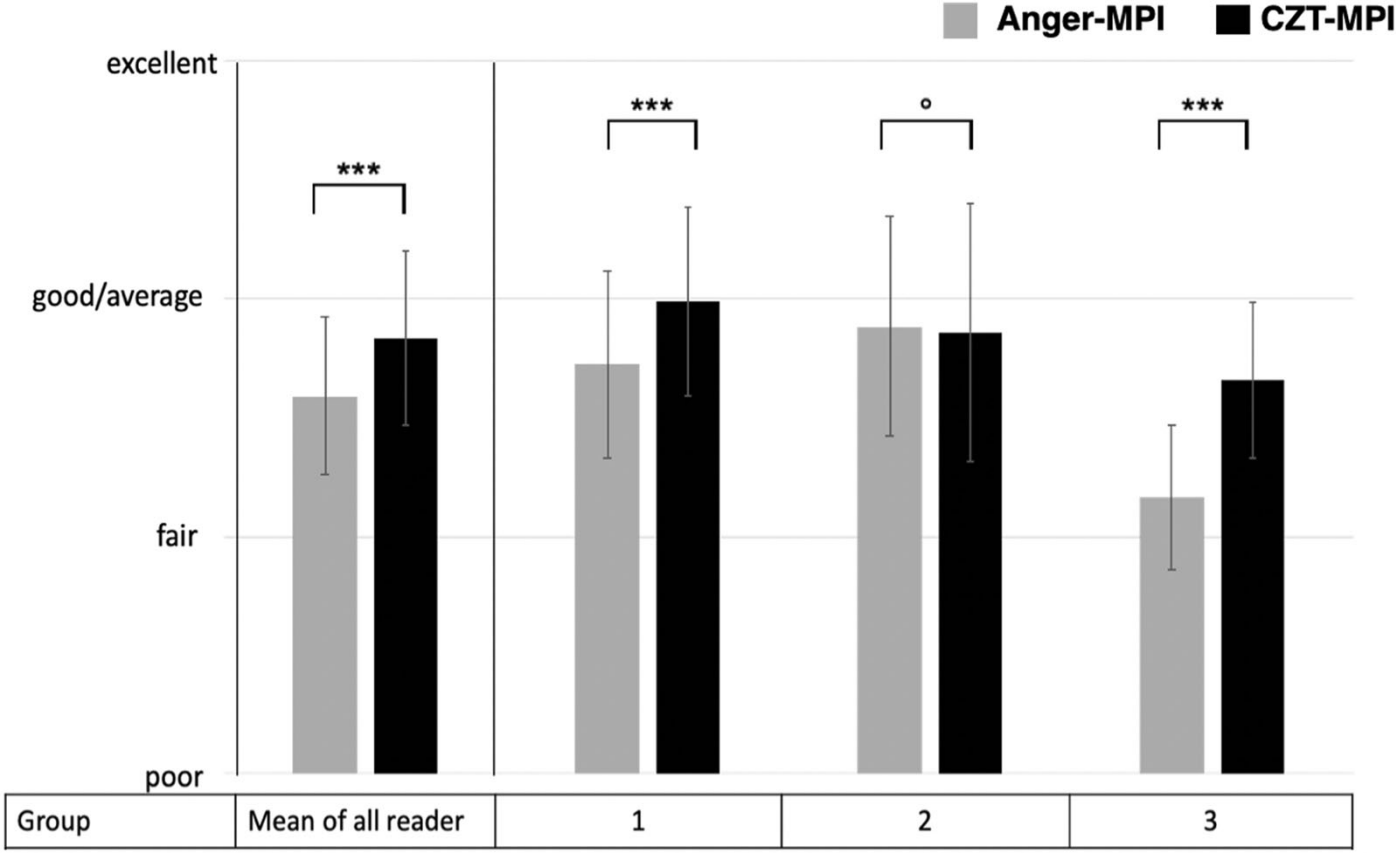
Image quality of conventional SPECT vs. CZT-SPECT in the groups of readers with varying experience. Bars represent mean image quality. Group 1: readers experienced with conventional SPECT and CZT-SPECT; Group 2: readers only experienced with conventional SPECT; Group 3: readers with little or no experience in interpretation of SPECT MPI

**Figure 3:**
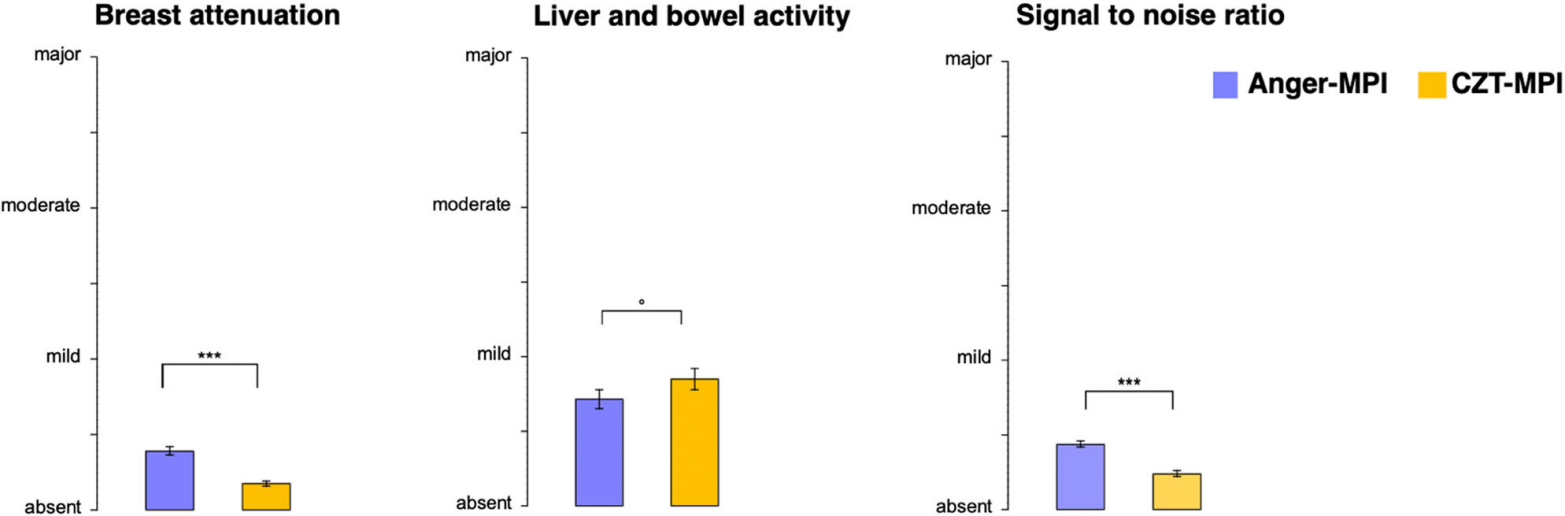
Mean artifacts MPI, myocardial perfusion imaging; *CZT* cadmium-zinc-telluride

Image quality of the D-SPECT was rated significantly better compared to conventional SPECT cameras (2.8 ± 0.36 vs. 2.6 ± 0.33, *P* < 0.05, Figure 2). In addition, significantly less breast attenuation was observed in the scans acquired with the CZT camera (1.2 ± 0.14 vs. 1.4 ± 0.26, *P* < 0.05), regardless of reader experience (Figure 3). When examining the relationship between breast attenuation and gender (N = 49 male, N = 34 female), the overall differences for CZT-MPI were not significant (1.19 ± 0.14 for males vs. 1.17 ± 0.14 for females, *P *= 0.6). Higher breast attenuation was observed in females when examined with Anger-MPI (1.33 ± 0.18 for males vs. 1.48 ± 0.33 for females), yet this difference was not significant (*P*=0.052). A significant difference in breast attenuation between female and male patients was observed only in MPI findings of the Anger cameras and only by readers who only had experience with conventional SPECT cameras (Group 2, 1.3 ± 0.21 vs. 1.63 ± 0.45, *P* < 0.05). Another possible source of artifacts is liver and bowel activity. The CZT camera exhibited slightly higher liver and bowel activity, but no significant difference for any reader group was observed (1.85 ± 0.65 vs. 1.71 ± 0.59, *P*=  0.073, Figure 3). When comparing liver and bowel activity in correlation with the time interval between the first and second stress scan, a significant negative correlation was observed (*pτ* < 0.05). This observation was made by the experienced readers (groups 1 and 2), but not by the inexperienced readers (group 3, *P *=0.074). Although the stress MPI was performed as the first procedure in 80% of the patients on the CZT camera, and a significant negative correlation between time and liver and bowel artifacts was confirmed, this potential artifact was not significantly higher for the CZT camera compared to conventional SPECT (1.85 ± 0.65 vs. 1.71 ± 0.59, *P *= 0.073, Figure 3). In addition, visual assessment of the CZT camera images showed a higher signal-to-noise ratio than that of the conventional cameras.

### Perfusion abnormalities

**Figure 4:**
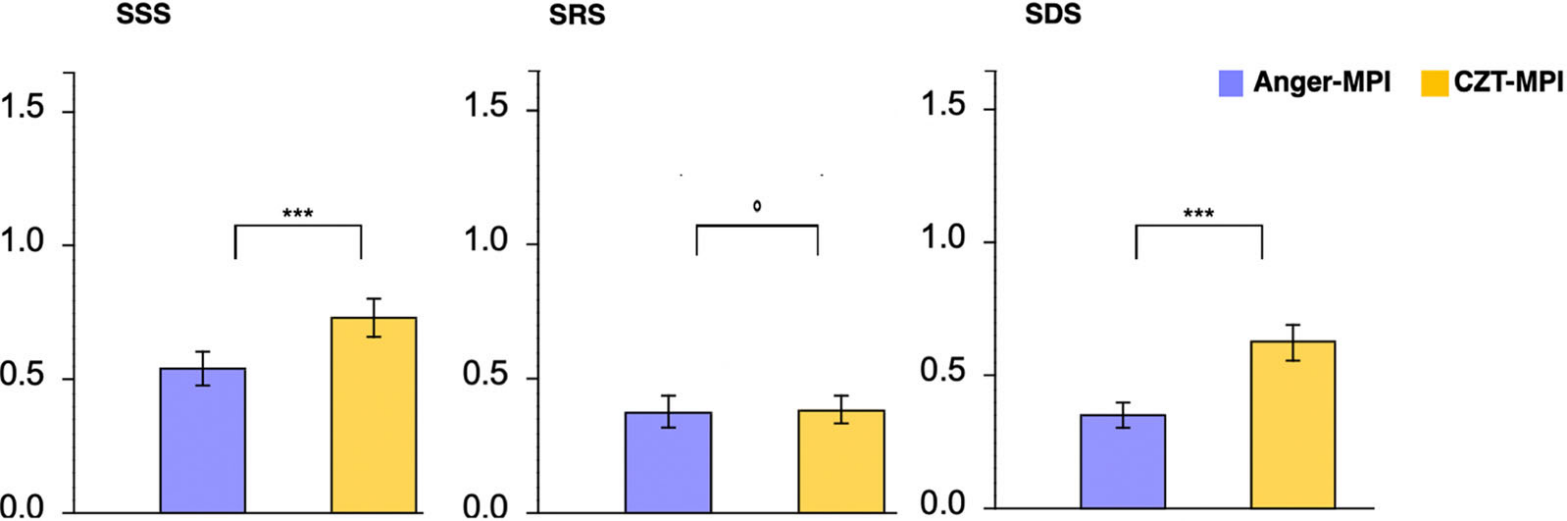
Scores based on 17 segment model of the American heart association (AHA). *SSS* summed stress score, *SRS* summed rest score, *SDS* summed difference score, *MPI* myocardial perfuion imaging, *CZT* cadmium-zinc-telluride

Regarding the final diagnosis, all reader groups observed a significantly higher rate of abnormalities in CZT-MPI (0.5 ± 0.32 vs. 0.37 ± 0.30, *P* < 0.05). This correlation was also apparent with significantly higher mean SSS and SDS scores in all groups on CZT-MPI (SSS: 0.73 ± 0.63 vs. 0.54 ± 0.60, *P *= 0. 0.00028*, *P* < 0.05, SDS: 0.63 ± 0.62 vs. 0.36 ± 0.47, *P *= 0.00007*, *P* < 0.05). Mean SRS scores were not significantly different (0.39 ± 0.50 vs. 0.38 ± 0.53, *P *= 0.70, Figure 4). Readers experienced with both camera systems (group 1) had significantly lower SSS and SDS than the other groups, regardless of the camera system used (SSS: 0.42 ± 0.63 CZT vs. 0.28 ± 0.55 conventional, *P *< 0.05, SDS: 0.38 ± 0.63 CZT vs. 0.12 ± 0.27 conventional, *P *< 0.05). In contrary, inexperienced readers (group 3) reached the highest SSS (0.97 ± 0.73 CZT vs. 0.77 ± 0.73 conventional, *P *= 0.0071*, *P* < 0.05).

### Inter-rater agreement and interpretative certainty

**Figure 5:**
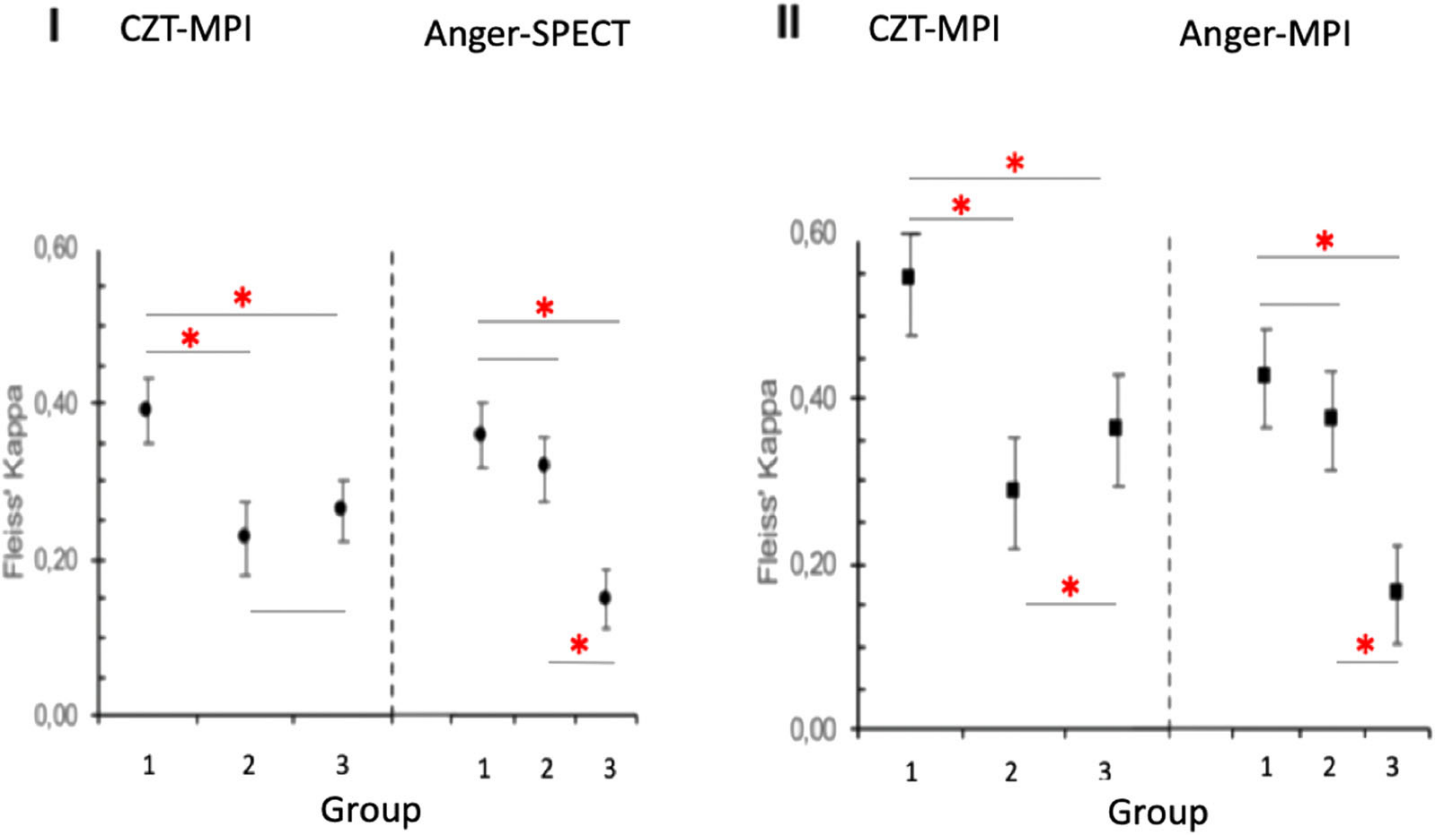
Inter-rater agreement. Inter-rater agreement was analyzed with the Fleiss' Kappa statistics. I: Inter-rater agreement regarding all answers for diagnosis (normal, ischemia, mixed ischemia/scar and scar); II: Inter-rater agreement regarding normal versus abnormal findings (normal versus all other answers). The kappa value is shown as a point with the respective two-sided confidence interval of 95%. Interpretation: < 0.00 = Poor agreement; 0.00 to 0.20 = Slight agreement; 0.21 to 0.40 = fair agreement; 0.41 to 0.60 = Moderate agreement; 0.61 to 0.80 = Substantial agreement; 0.81 to 1.00 = Almost perfect agreement. The red asterisk indicates statistical significance (*P* ≤ 0.05)

Readers with experience with both camera systems (group 1) reached a significantly higher degree of inter-rater agreement in comparison to reader groups 2 and 3 (CZT-MPI 0.54, Anger-MPI 0.49, *P* < 0.05). Group 2 showed significantly lower inter-rater agreement in CZT-MPI in comparison to Anger-MPI (0.23 vs. 0.37 in diagnosis and 0.29 vs. 0.43 in abnormality, *P* < 0.05). Group 3, with little or no experience in MPI, achieved a significantly higher degree of agreement in CZT-MPI than with the Anger-MPI (0.26 vs. 0.17 in diagnosis and 0.36 vs. 0.19 in abnormality, *P* < 0.05), respectively. Regardless of the modality, the inter-rater agreement of group 3 was always lower compared to group 1 (Figure 5).

For the assessment of interpretative certainty, the answers were divided into three columns: equivocal, probable (normal or abnormal), and definite (normal or abnormal). Higher values indicated greater interpretative certainty regarding final diagnosis based purely on image interpretation. Experienced readers (groups 1 and 2) had significantly higher interpretative certainty than readers with little or no experience in MPI interpretation (group 3), (2.5 vs. 2.2 in CZT-MPI, 2.43 vs. 2.14 in Anger-MPI, *P* < 0.05). Mean overall diagnostic certainty was comparable for both camera systems (2.34 vs. 2.39, *P* = 0.38).

### Sensitivity and specificity

In patients undergoing coronary angiography, eight patients had relevant coronary artery stenosis, defined as a ≥ 70% stenosis of at least one coronary artery, and two patients had normal angiograms. A negative control group (N = 10) was defined as patients with no relevant coronary artery stenosis in coronary angiography (N = 2) as well as patients who had intermediate pretest probability for CAD, yet no indication for a coronary angiography after revision of all non invasive examinations and available data and exclusion of CAD in interdisciplinary consensus (N = 8). Overall, the D-SPECT showed a higher sensitivity compared to conventional SPECT cameras (88% versus 63%, *P* <0.05, CI-CZT 0.47-0.99, CI-conv. 0.25 to 0.91). Specificity of the CZT camera was significantly lower than of the conventional cameras (40% versus 100%, *P* < 0.05, CI-CZT. 0.12 to 0.74, CI-conv. 0.69 to 1.00). Higher rates of false positive evaluations resulted in overall reduced accuracy for CZT MPI, as shown in Table 2 (61% versus 83%, *P* <0.05).

**Table 2:** Sensitivity and specificity

	Camera	Sensitivity (%)	Specificity (%)	Accuracy (%)
Group 1	Conventional	37.5	100	72
	CZT	62.5	60	61
Group 2	Conventional	75	80	78
	CZT	87.5	30	56
Group 3	Conventional	87.5	60	72
	CZT	87.5	20	50
Overall	Conventional	62.5	100	83
	CZT	87.5	40	61

Group 1: readers experienced with both conventional SPECT cameras and a CZT-SPECT camera; Group 2: readers only experienced with conventional SPECT cameras; Group 3: readers with little or no experience in MPS interpretation

## Discussion

Novel CZT-MPI systems have many advantages over conventional Anger-MPI, a concept which has not changed significantly over more than five decades, however, the influence on inter-reader reproducibility and diagnostic certainty compared to conventional SPECT systems has been insufficiently investigated. The aim of this study was to compare two technologically different camera systems in the identical patient cohort with the use of a standardized questionnaire in a multicentric setting and to study inter-rater reproducibility, reader experience effects, and diagnostic accuracy of the different MPI technologies among readers from the field of nuclear cardiology with different levels of experience in MPI interpretation.

Better image quality of the CZT camera was confirmed and is technically due to its significantly enhanced sensitivity of count detection compared to conventional Anger cameras as previously confirmed in phantom models by Zoccarato et al.^24^. In addition, heart-specific iterative reconstruction methods, considering the collimation geometry of CZT-MPI, improve image contrast and reduce noise level as confirmed in this study. The good quality of CZT images is also confirmed with less breast attenuation observed by all reader groups, regardless of underlying experience. When compared with coronary angiography, higher sensitivity (87.5% vs 62.5%) and significantly lower specificity for the CZT camera were observed (40% vs 100%). In the meta-analysis by Nudi et al.^8^ comparing CZT-MPI and conventional Anger-MPI, breast attenuation and obesity were discussed as possible causes for the reduced specificity of CZT technology as extra-cardiac soft tissue activity can still hamper image quality and contrast-to-noise ratio possibly generating false positive results arising especially in inferior and inferolateral segments.^9,10^ Some studies suggest greater diagnostic accuracy in combined supine and prone CZT MPI without adversely affecting its sensitivity.^25^ However, this comes at the cost of extended examination time and is therefore recommended in the case of inferolateral ischemia in patients with a history of previous inferolateral MI.^26^ Therefore, routine prone imaging in CZT-MPI and adequate training and learning curve of the technologists, especially with patient positioning, are key in yielding the best images this technology can provide. Lack of experience of the technologists at the time of introduction of the D-SPECT could have contributed to the low specificity of this camera in our study.

Although the stress MPI with the CZT camera was performed as first procedure in 80% of the patients, artifacts due to liver and bowel activity were not considered significantly more serious for the CZT camera. It is noticeable that the experience of the reader plays a significant role in the interpretation of artifacts as such. All inexperienced readers (group 3) achieved a significantly lower specificity with the CZT camera (mean specificity of 20%). Overall, they observed significantly more abnormalities in the CZT-MPI findings than in conventional Anger-MPI findings. The tendency for overestimation of the disease was shown repeatedly in questions about diagnosis and in assessments with significantly higher SSS and SDS reached by this group (Figure 4). Fiechter et al.^6^ discussed that due to the improved spatial resolution of the CZT detectors, better visualization of non-serious perfusion defects in the absence of significant CAD can also impair the specificity of CZT-based camera systems as also observed in this study. A large multicenter study evaluating fractional flow reserve (FFR) in coronary lesions found that the majority of coronary lesions with a narrowing of 50–70% have no hemodynamic relevance.^27^ Although the degree of agreement between conventional Anger-MPI and FFR measurements has been well investigated, to our knowledge, this is not the case for CZT-MPI.^28^ Therefore, further investigation of CZT-MPI in correlation with the FFR will certainly be required in the future.

Significantly higher Inter-rater agreement in the reader group with experience with CZT based cameras (group 1) compared to the rest of the groups (2 and 3) with no significant experience with CZT-MPI interpretation illustrates that regardless of the underlying experience, dedicated training with such systems is required to avoid over- or underestimation of results. Importantly, for optimal image acquisition technologists should also undergo training and acquire experience regarding optimal patient positioning in CZT-MPI.^29^

## Limitations of the Study

It is important to emphasize that a purely image-based, visual evaluation of the myocardial perfusion scintigraphy can be considered as a study limitation. This does not reflect the clinical reality, as various clinical parameters play a role in the diagnostic work-up of CAD and everyday decision-making. However, as we were interested in a direct head-to-head comparison of imaging systems, we considered this unavoidable. The use of a standardized reading template, the anonymization and randomization of the modality of the examination (CZT- vs. Anger-MPI) and the multicentric approach helped increase the power of this study and reduce familiarity bias. Another limitation in the use of a single cohort and sequential double examination is the extra cardiac clearance pattern and the limited but nevertheless existing decay of radiotracer with time between the examinations. However, a study with separate examination protocols for the same patient cohort on different days is not justifiable due to the unnecessary radiation exposure. In the present study, no attenuation corrected images (either by CT or by alternative positioning of the patient) were provided to the readers. This came at the risk of attenuation artifacts being misinterpreted as perfusion abnormalities However, routine attenuation correction (e.g., by CT or by routine repositioning of the patient) is not performed in our clinic, so that attenuation-corrected images would only be available for a small number of patients. Therefore, we decided to compare the images primarily created in the clinic with standard positioning of the patient, also in order to better capture the different image properties of the respective camera systems.

## Conclusion

CZT-MPI delivers higher diagnostic sensitivity compared to conventional Angel-MPI whilst exhibiting significantly lower specificity. Our investigation has shown that the reduced accuracy of CZT-MPI due to low specificity compared to Anger-MPI—especially achieved by nuclear cardiologist with no previous experience in CZT-MPI—illustrates the necessity of a dedicated training. The importance of a differentiated evaluation and interpretation of the results of non-invasive diagnostics in everyday decision-making and clinical practice was demonstrated.

## New Knowledge Gained

CZT-MPI displays superior image quality and lower susceptibility for artifacts. Reader experience plays a major role in the interpretation of SPECT images, especially when acquired by camera systems with high diagnostic sensitivity. Therefore, when introducing a CZT-detector-based camera, dedicated training of the medical staff is recommended.

## Supplementary Information

Below is the link to the electronic supplementary material.

Supplementary file1 (PPTX 8306 KB)

Supplementary file2 (MP3 7589 KB)

## Abbreviations

MPI: Myocardial perfusion imaging
SPECT: Single photon emission computed tomography
CZT: Cadmium-zinc-telluride
CAD: Coronary artery disease
AHA: American Heart Association
Tc-99m: Technetium-99m
MIBI: Sestamibi
MBq: Megabecquerel
AC: Attenuation correction
EF: Ejection fraction
