# Supplementary material for: Multi-center study of inter-rater reproducibility, image quality, and diagnostic accuracy of CZT versus conventional SPECT myocardial perfusion imaging
Source: J Nucl Cardiol. 2022 Jul 7;30(2):528–39. doi: 10.1007/s12350-022-03054-w (PMC10125926; doi:10.1007/s12350-022-03054-w)
Supplement: Supplementary file 1 — Supplementary file1 (PPTX 8306 KB) [file 12350_2022_3054_MOESM1_ESM.pptx]

## Slide 1
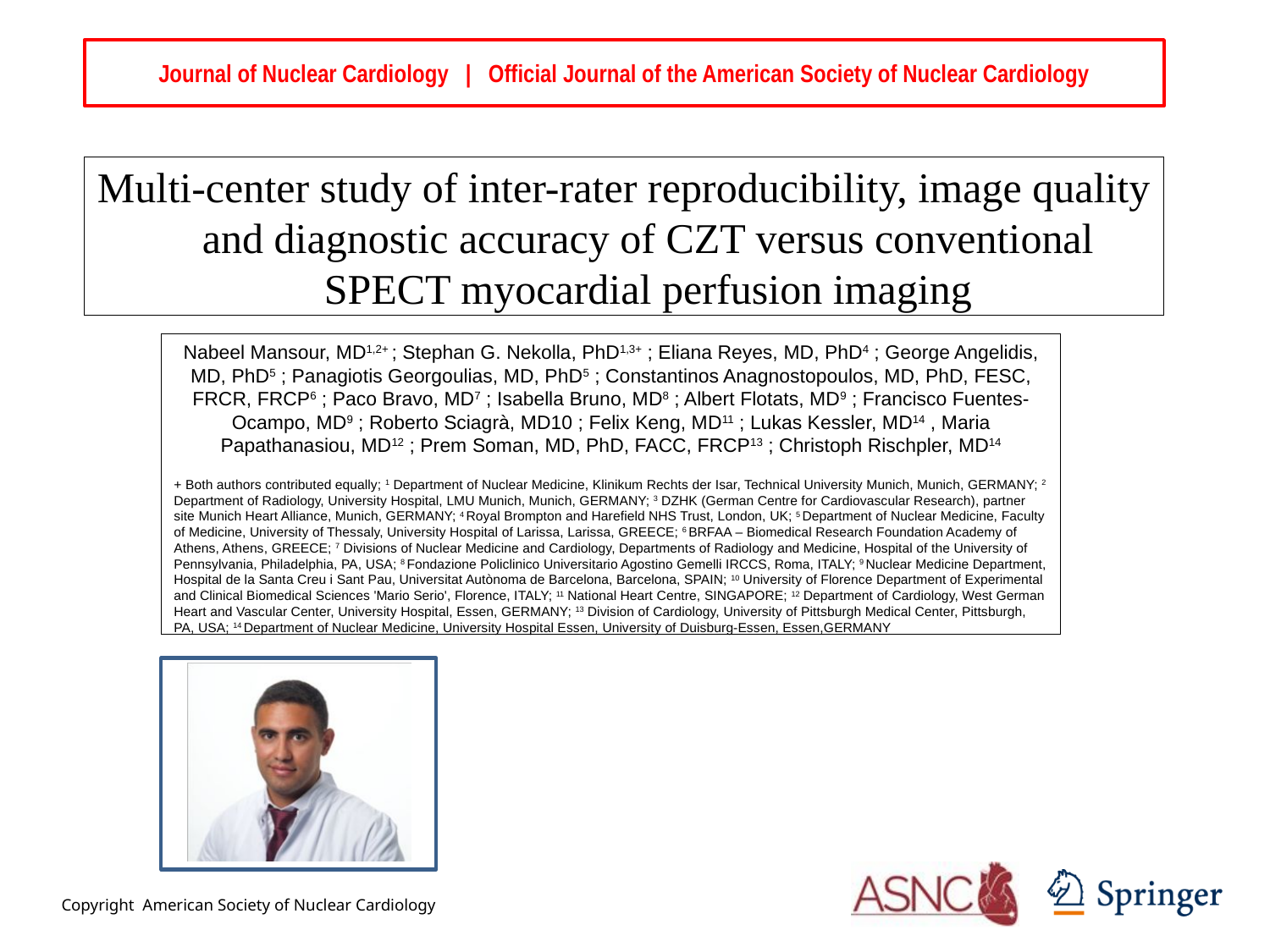

Journal of Nuclear Cardiology | Official Journal of the American Society of Nuclear Cardiology
# Multi-center study of inter-rater reproducibility, image quality and diagnostic accuracy of CZT versus conventional SPECT myocardial perfusion imaging
Nabeel Mansour, MD1,2+ ; Stephan G. Nekolla, PhD1,3+ ; Eliana Reyes, MD, PhD4 ; George Angelidis, MD, PhD5 ; Panagiotis Georgoulias, MD, PhD5 ; Constantinos Anagnostopoulos, MD, PhD, FESC, FRCR, FRCP6 ; Paco Bravo, MD7 ; Isabella Bruno, MD8 ; Albert Flotats, MD9 ; Francisco Fuentes-Ocampo, MD9 ; Roberto Sciagrà, MD10 ; Felix Keng, MD11 ; Lukas Kessler, MD14 , Maria Papathanasiou, MD12 ; Prem Soman, MD, PhD, FACC, FRCP13 ; Christoph Rischpler, MD14
+ Both authors contributed equally; 1 Department of Nuclear Medicine, Klinikum Rechts der Isar, Technical University Munich, Munich, GERMANY; 2 Department of Radiology, University Hospital, LMU Munich, Munich, GERMANY; 3 DZHK (German Centre for Cardiovascular Research), partner site Munich Heart Alliance, Munich, GERMANY; 4 Royal Brompton and Harefield NHS Trust, London, UK; 5 Department of Nuclear Medicine, Faculty of Medicine, University of Thessaly, University Hospital of Larissa, Larissa, GREECE; 6 BRFAA – Biomedical Research Foundation Academy of Athens, Athens, GREECE; 7 Divisions of Nuclear Medicine and Cardiology, Departments of Radiology and Medicine, Hospital of the University of Pennsylvania, Philadelphia, PA, USA; 8 Fondazione Policlinico Universitario Agostino Gemelli IRCCS, Roma, ITALY; 9 Nuclear Medicine Department, Hospital de la Santa Creu i Sant Pau, Universitat Autònoma de Barcelona, Barcelona, SPAIN; 10 University of Florence Department of Experimental and Clinical Biomedical Sciences 'Mario Serio', Florence, ITALY; 11 National Heart Centre, SINGAPORE; 12 Department of Cardiology, West German Heart and Vascular Center, University Hospital, Essen, GERMANY; 13 Division of Cardiology, University of Pittsburgh Medical Center, Pittsburgh, PA, USA; 14 Department of Nuclear Medicine, University Hospital Essen, University of Duisburg-Essen, Essen,GERMANY
Copyright American Society of Nuclear Cardiology

## Slide 2
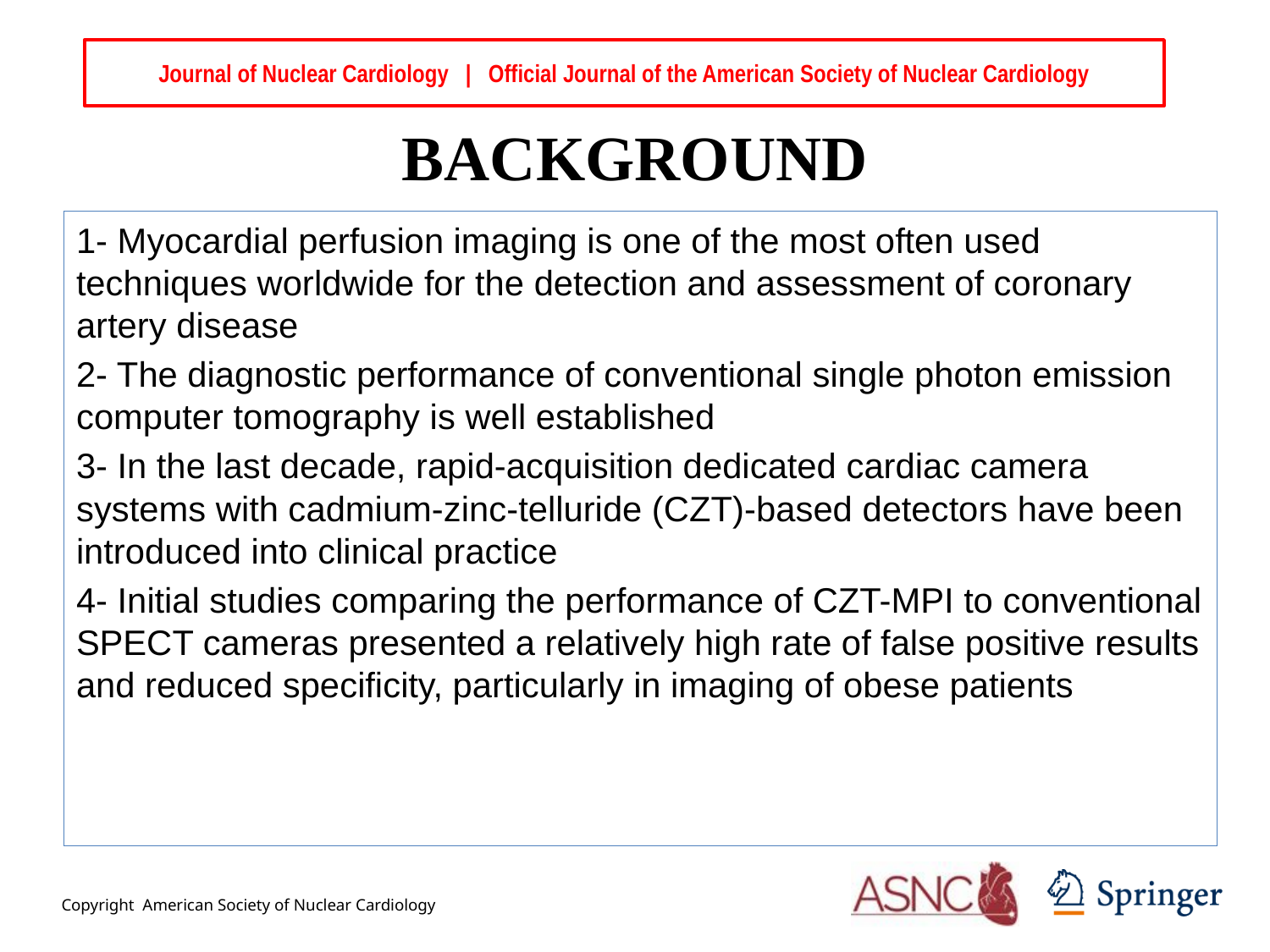

Journal of Nuclear Cardiology | Official Journal of the American Society of Nuclear Cardiology
# BACKGROUND
1- Myocardial perfusion imaging is one of the most often used techniques worldwide for the detection and assessment of coronary artery disease
2- The diagnostic performance of conventional single photon emission computer tomography is well established
3- In the last decade, rapid-acquisition dedicated cardiac camera systems with cadmium-zinc-telluride (CZT)-based detectors have been introduced into clinical practice
4- Initial studies comparing the performance of CZT-MPI to conventional SPECT cameras presented a relatively high rate of false positive results and reduced specificity, particularly in imaging of obese patients
Copyright American Society of Nuclear Cardiology

## Slide 3
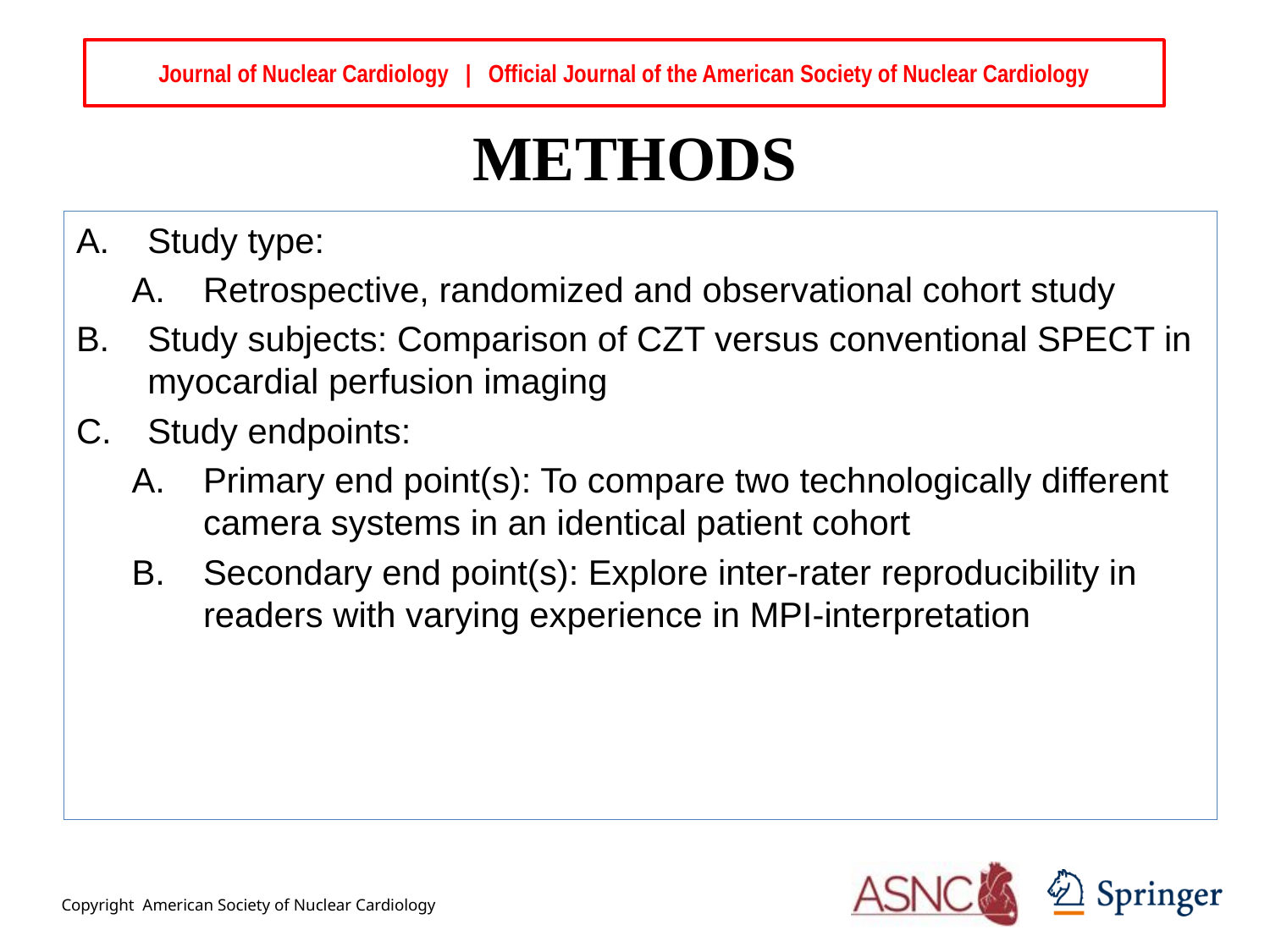

Journal of Nuclear Cardiology | Official Journal of the American Society of Nuclear Cardiology
# METHODS
Study type:
Retrospective, randomized and observational cohort study
Study subjects: Comparison of CZT versus conventional SPECT in myocardial perfusion imaging
Study endpoints:
Primary end point(s): To compare two technologically different camera systems in an identical patient cohort
Secondary end point(s): Explore inter-rater reproducibility in readers with varying experience in MPI-interpretation
Copyright American Society of Nuclear Cardiology

## Slide 4
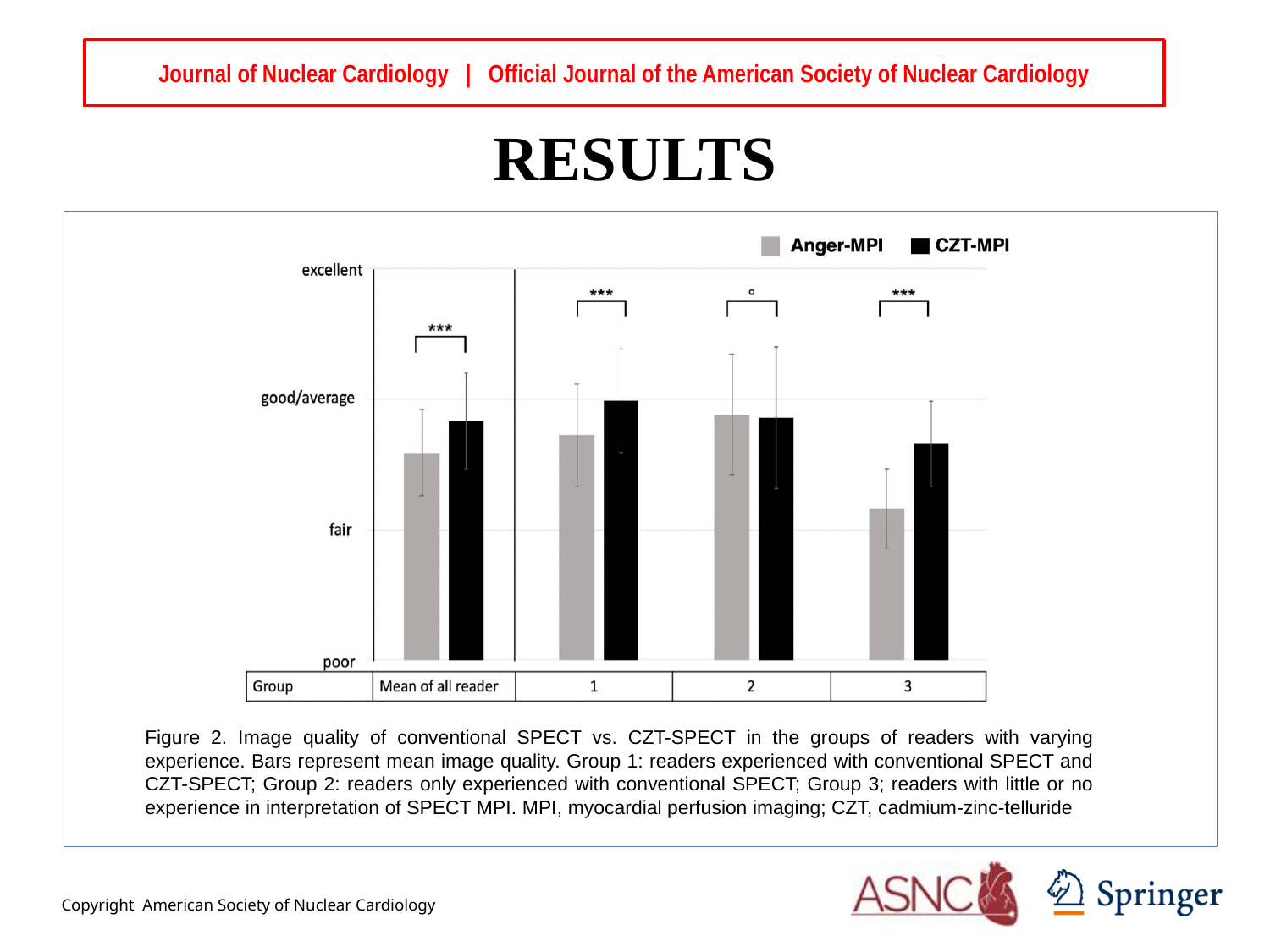

Journal of Nuclear Cardiology | Official Journal of the American Society of Nuclear Cardiology
# RESULTS
Figure 2. Image quality of conventional SPECT vs. CZT-SPECT in the groups of readers with varying experience. Bars represent mean image quality. Group 1: readers experienced with conventional SPECT and CZT-SPECT; Group 2: readers only experienced with conventional SPECT; Group 3; readers with little or no experience in interpretation of SPECT MPI. MPI, myocardial perfusion imaging; CZT, cadmium-zinc-telluride
Copyright American Society of Nuclear Cardiology

## Slide 5
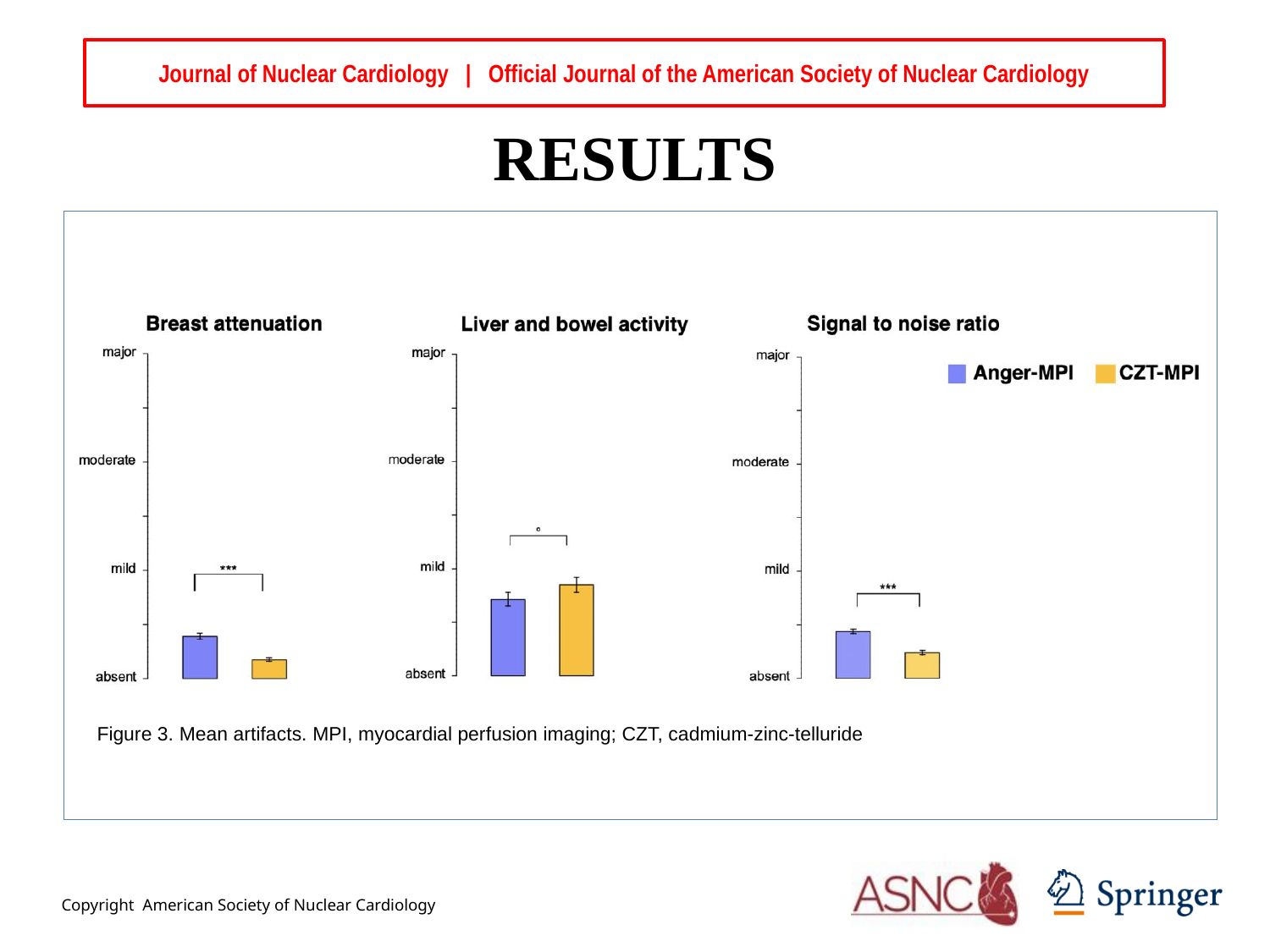

Journal of Nuclear Cardiology | Official Journal of the American Society of Nuclear Cardiology
# RESULTS
Insert a key table or a key figure
If figure, insert legend
Figure 3. Mean artifacts. MPI, myocardial perfusion imaging; CZT, cadmium-zinc-telluride
Copyright American Society of Nuclear Cardiology

## Slide 6
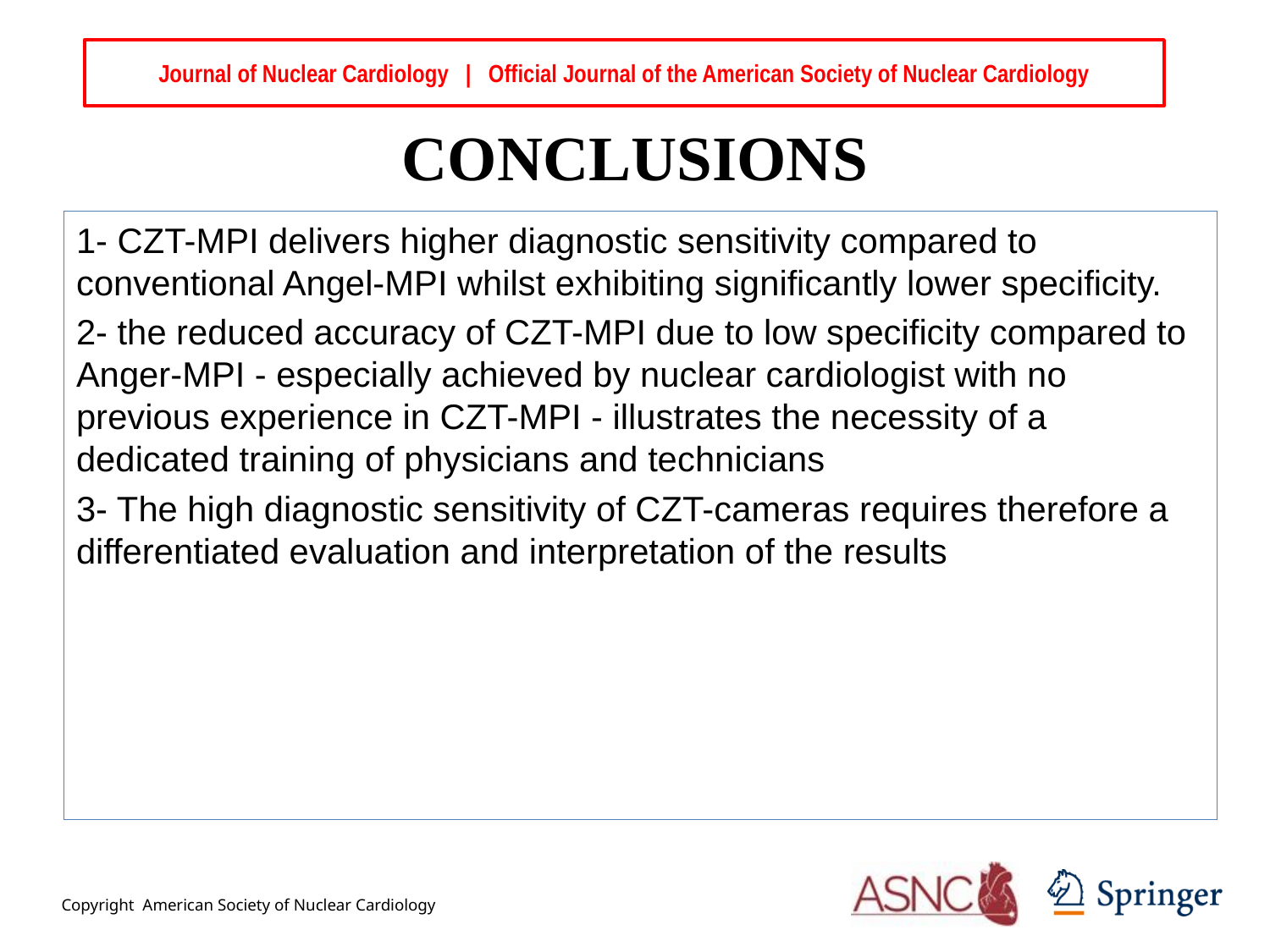

Journal of Nuclear Cardiology | Official Journal of the American Society of Nuclear Cardiology
# CONCLUSIONS
1- CZT-MPI delivers higher diagnostic sensitivity compared to conventional Angel-MPI whilst exhibiting significantly lower specificity.
2- the reduced accuracy of CZT-MPI due to low specificity compared to Anger-MPI - especially achieved by nuclear cardiologist with no previous experience in CZT-MPI - illustrates the necessity of a dedicated training of physicians and technicians
3- The high diagnostic sensitivity of CZT-cameras requires therefore a differentiated evaluation and interpretation of the results
Copyright American Society of Nuclear Cardiology
